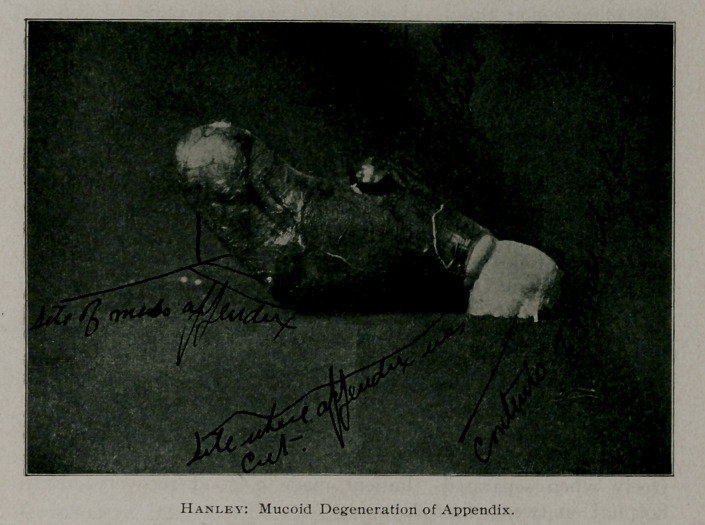# Report of Four Cases

**Published:** 1906-01

**Authors:** L. G. Hanley

**Affiliations:** Buffalo, N. Y.; Surgeon to the Buffalo Hospital of the Sisters of Charity; Surgeon to the Emergency Hospital; Chief Obstetrician to St. Mary’s Infant and Maternity Hospital; Consulting Obstetrician to Erie County Hospital; Clinical Professor of Obstetrics, University of Buffalo


### CLINICAL REPORTS.

### Report of Four Cases.

By L. G. HANLEY, M. D., Ph. D., Buffalo, N. Y.

Surgeon to the Buffalo Hospital of the Sisters of Charity; Surgeon to the Emergency Hospital; Chief Obstetrician to St. Mary's Infant and Maternity Hospital; Consulting Obstetrician to Erie County Hospital; Clinical Professor of Obstetrics, University of Buffalo.

### MUCOID DEGENERATION OF MUCOUS MEMBRANE OF THE APPENDIX

Ed. B. Age 37. Boiler maker. U. S. Parents dead. Father killed. Mother died of pulmonary tuberculosis. Three sisters and one brother died of tuberculosis. Never had any sickness until twelve years ago when he had "inflammation of the bowels, with obstruction." At that time, he reports, that his bowels did not move for nine days and his physicians thought that he would die.

Hanley: Mucoid Degeneration of Appendix.

One month from time of this attack he resumed work and has continued to follow his avocation up to three weeks prior to coming under my care, which was November 9, 1905. During those three weeks he had pain in right side in region of appendix, eructations of gas and distress after eating and has lost in weight during these three weeks, twenty-two pounds. He preferred not to eat rather than suffer pain.

Patient prepared for operation. Kidneys, lungs and heart in good condition. Pulse and temperature normal. Leukocyte count 7,000. There is pain upon pressure two inches below normal portion of appendix, and one inch toward median line. On opening the abdomen a mass three and one-half inches long and one and one-quarter inches thick, which proves to be the appendix, was delivered. The meso-appendix with cord-like adhesions encircled the entire mass. Specimen removed and patient put to bed. Left hospital sixteen days after operation.

The report of examination from the Laboratory of Pathology, University of Buffalo, shows:

"The appendix is dilated and filled with a mucoid substance, which responds chemically to mucin. There is no evidence of carcinomatous infiltration of the coats of the appendix, but there is some inflammatory change with congestion of the blood vessels. It is my opinion that the condition is a mucoid degeneration of the mucus membrane of the appendix." CHARLES A. BENTZ.

### LOCALISED PERITONITIS CONSEQUENT UPON STRANGULATION OF OMENTUM DUE TO INJURY. APPENDIX SECONDARILY INVOLVED.

Charles Z. Age 33. Fireman. Has always been well until three days before operation, the date of which was October 13, 1905. He thinks he might have hurt himself sliding down a pole in the fire house. Pulse 90, temperature 101°. Leukocytosis 16,000. There is pain upon the slightest pressure and spasm of muscles of right side, extending from Pouparts ligament to ninth intercostal space. On opening abdomen a piece of omentum, 6 by 5 inches and 1½ inches thick, adherent to peritoneum and ileum and cecum in a strangulated semigangrenous condition, was found. The appendix lay posterior to this mass and was greatly inflamed from contact with diseased omentum. The serosa of appendix was very much engorged but the mucosa seemed normal. The peritoneum, where the omentum was attached, and the serous surface of ileum bled freely when separated. Patient made a good recovery. Left hospital on twenty-first day.

John S. Age 43. U. S. Family history good. Personal history good until one month ago when strained himself, lifting. The pain he experienced at that time did not hinder him from following his occupation of teamster. On November 15, 1905, pain in his side became so severe that he was obliged to go to bed. Again resumed his work the next day. On November 19, 1905, came under my care. Examination shows localised spasm of muscles extending from Pouparts ligament to median line and to ninth intercostal space. Painful upon pressure and so severe that patient is unable to stand and only finds relief in lying upon his back with legs and thighs flexed.

Pulse 84; temperature 99; Leukocyte count 8,000. On opening abdomen a piece of omentum 4 by 3 inches square was found adhered to cecum and peritoneum, strangulated and semigangrenous. The appendix was four inches long, thickened and the serosa very much inflamed from contact with diseased omentum. Appendix and mass removed. Patient's condition for first four days after operation was normal as regards temperature, pulse, the taking of nourishment, secretion of kidneys, movements of bowels, etc., but on the sixth day he developed a delerium the nature of nimia bibens, though his history showed that he was not in the habit of using intoxicants to excess, and in the absence of nurse jumped from the third story of hospital, through window, breaking glass, and fell a distance of 40 feet. Though he broke the glass he did not break any bones and from a few cuts on face and a good shaking up made a good recovery. Wound in abdomen healed by first intention. Left hospital in twenty days.

HERNIOTOMY, SAC CONTENTS, THE APPENDIX.

Mr. B. Age 59. Family history good. Carpenter. Always been well until two years ago when by straining in attempting to lift a heavy box he developed a right inguinal hernia. He has never worn a truss and claims that he could easily replace a swelling that would appear by making a little pressure with his hand. Two months before coming under my care he was unable to work and found that he was unable to reduce hernia when it would descend. For hours he would lie upon his back and hernia would be reduced with difficulty. Pain that was not severe at first has greatly increased until he is unable to stand with comfort and only feels good when lying upon his back.

Examination shows rather small sac easily reduced by taxis. Patient operated September 18, 1905. Sac contains a substance about four inches long, hard, and attached to sac. This proves to be the appendix which was separated from sac and removed. It was free from any fecal substance or foreign body and its lumen was entirely obliterated (appendix obliterans). Herniotomy performed. Patient left hospital in two weeks.

428 PORTER AVENUE.

## Figures and Tables

**Figure f1:**